# Cognition and bimanual performance in children with unilateral cerebral palsy: protocol for a multicentre, cross-sectional study

**DOI:** 10.1186/s12883-018-1070-z

**Published:** 2018-05-08

**Authors:** Brian Hoare, Michael Ditchfield, Megan Thorley, Margaret Wallen, Jenny Bracken, Adrienne Harvey, Catherine Elliott, Iona Novak, Ali Crichton

**Affiliations:** 1 Victorian Paediatric Rehabilitation Service, Monash Children’s Hospital, 246 Clayton Rd, Clayton, VIC 3168 Australia; 2 School of Occupational Therapy, La Trobe University, Bundoora, VIC 3168 Australia; 3 Department of Paediatrics, Monash University, Clayton, VIC 3168 Australia; 4 Department of Diagnostic Imaging, Monash Children’s Hospital, 246 Clayton Road, Clayton, VIC 3168 Australia; 5 Queensland Paediatric Rehabilitation Service, Lady Cilento Children’s Hospital, South Brisbane, QLD 4101 Australia; 6 School of Allied Health, Australian Catholic University, North Sydney, NSW 2060 Australia; 7 Department of Diagnostic Imaging, Royal Children’s Hospital, 50 Flemington Rd, Parkville, Victoria, 3052 Australia; 8 Developmental Disability and Rehabilitation Research, Murdoch Children’s Research Institute, Parkville, VIC 3052 Australia; 9 School of Occupational Therapy and Social Work, Curtin University, Bentley, 6102 Western Australia Australia; 10 Cerebral Palsy Alliance, Child and Adolescent Health, The University of Sydney, PO Box 6427, Frenchs Forest, NSW 2086 Australia; 11 Department of Paediatric Rehabilitation, Princess Margaret Hospital, Washington, WA Australia

**Keywords:** Cerebral palsy, Children, Cognition, Upper limb, Bimanual performance, Occupational therapy, Neuropsychology

## Abstract

**Background:**

Motor outcomes of children with unilateral cerebral palsy are clearly documented and well understood, yet few studies describe the cognitive functioning in this population, and the associations between the two is poorly understood. Using two hands together in daily life involves complex motor and cognitive processes. Impairment in either domain may contribute to difficulties with bimanual performance. Research is yet to derive whether, and how, cognition affects a child’s ability to use their two hands to perform bimanual tasks.

**Methods/Design:**

This study will use a prospective, cross-sectional multi-centre observational design. Children (aged 6–12 years) with unilateral cerebral palsy will be recruited from one of five Australian treatment centres. We will examine associations between cognition, bimanual performance and brain neuropathology (lesion type and severity) in a sample of 131 children. The primary outcomes are: Motor - the Assisting Hand Assessment; Cognitive - Executive Function; and Brain – lesion location on structural MRI. Secondary data collected will include: Motor - Box and Blocks, ABILHAND- Kids, Sword Test; Cognitive – standard neuropsychological measures of intelligence. We will use generalized linear modelling and structural equation modelling techniques to investigate relationships between bimanual performance, executive function and brain lesion location.

**Discussion:**

This large multi-centre study will examine how cognition affects bimanual performance in children with unilateral cerebral palsy. First, it is anticipated that distinct relationships between bimanual performance and cognition (executive function) will be identified. Second, it is anticipated that interrelationships between bimanual performance and cognition will be associated with common underlying neuropathology. Findings have the potential to improve the specificity of existing upper limb interventions by providing more targeted treatments and influence the development of novel methods to improve both cognitive and motor outcomes in children with unilateral cerebral palsy.

**Trial registration:**

ACTRN12614000631606; Date of retrospective registration 29/05/2014.

## Background

Cerebral palsy (CP) is the most common cause of physical disability in childhood and is attributed to non-progressive disturbances that occur in the developing foetal or infant brain [1]. It is defined as “a group of permanent disorders of the development of movement and posture, causing activity limitation…” [1]. Consistent with this definition, the traditional focus of research in children with CP has been on exploring and evaluating methods to alter movement and posture to improve daily task performance [2]. Activity limitation however, is influenced by complex interactions between the environment, the task and multiple other child systems including sensation, perception, communication, behaviour and cognition [3, 4]. We propose that motor and cognitive impairments provide unique and related contributions to the ability of children to perform bimanual activities [5–7].

### Unilateral cerebral palsy

Unilateral CP, also known as hemiplegic CP, is characterised by a clinical pattern of unilateral motor impairment. It is the most common type of CP - 39% of people with CP in Australia present with unilateral CP [8]. The severity of motor impairment varies widely, depending on the site and severity of brain lesion [9, 10]. The functional impact of unilateral upper limb impairment has been the focus of extensive research undertaken to improve motor performance and independence with daily activities [11]. As a result, upper limb interventions such as constraint-induced movement therapy (CIMT) and bimanual therapy have strong evidence supporting effectiveness in children with unilateral CP [2]. However, not all children make clinically important change following these interventions [12]. We do not understand why a proportion of children with unilateral CP do not respond to evidence-based upper limb intervention [12]. As skilled task performance involves complex cognitive processes, it is reasonable to postulate that cognitive impairment may be associated with reduced ability of children to learn how to effectively use their two hands together to perform tasks. Identifying and better understanding the hidden impairments of children with unilateral CP, such as cognition, and their impact on task performance is important to more accurately tailor intervention. Understanding the common neuropathology and potential linkages between motor and cognitive phenotypes, and their impacts on bimanual performance, provides an opportunity for a more targeted and individualised approach to therapy. Ultimately, this will help to identify *what works best, for whom*.

### Early brain lesion and cognition in cerebral palsy

Lesion to the brain in CP occurs in the early stages of development, either in the prenatal, perinatal or early postnatal periods (up to 2 years) [13]. Early brain injury impacts concomitantly on motor and cognitive development and function [14], yet the impact is not uniformly seen across these domains. Further, cognitive impacts may be realised only later in childhood due to the protracted nature of cognitive development, relative to motor skill development. In particular, higher-level cognitive skills develop in parallel to the extended neurodevelopment of the prefrontal regions of the brain [15], beginning in infancy [16] and continuing through the pre-school years [17], middle childhood [18] and into adolescence [19].

### Cognitive profiles of children with cerebral palsy

#### Cognition

Cognition is not a unitary concept and is defined as “the mental action or process of acquiring knowledge and understanding through thought, experience, and the senses [20]”. Cognition includes a large number of individual interrelated and complex processes such as general intellectual function and executive functions. Until recently, cognitive function in children with CP has been broadly investigated and classified using measures of general intellectual function.

### Intellectual disability in children with cerebral palsy

Intellectual disability (ID) is defined as impairment of general mental abilities that impacts on adaptive functioning [21]. Data sourced from various national CP registers report a prevalence of ID in children with all types of CP that ranges from 17 to 60% [22–27]. The reason for this wide variation is likely due to different methods used to classify and measure ID [28], heterogeneity in the neuropathology and severity of CP and other associated comorbidities. In a recent study of 50 children with periventricular haemorrhagic infarction (PVHI) or perinatal arterial ischaemic stroke (PAIS), van Buuren et al. [29] reported a mean full-scale Intellectual Quotient (FSIQ) somewhat below the age mean but still within the average range. A FSIQ of 86 (95% CI 78–94) was documented for the PVHI group and 80 (95% CI 73–87) for the PAIS group. Similarly, in 46 children with unilateral CP (mean 11 years, 1 month, SD 2 years, 4 months) Bodimeade et al. report FSIQ scores of 84.95 (SD 14.65) for children with right unilateral CP and 86.75 (SD 17.95) for children with left unilateral CP. In both these studies Verbal IQ was better than Performance IQ, which is consistent with previous findings in children with early brain lesion [30–34].

Most studies report no association between FSIQ and lesion-related factors including side of lesion [29, 30, 35–37] and lesion location [29, 38]. Despite theories of neuroplasticity that provide theoretical support for increased flexibility of the young brain to reorganise in response to injury, poorer cognitive outcomes from very early lesions have been found when compared with lesions later in childhood [39, 40]. This suggests that early brain lesion may act to derail future brain maturation and development [41]. Due to the protracted development of cognitive abilities, cognitive impacts following early brain lesions emerge throughout development, and as such, some studies have documented slower gains over time compared with typically developing peers – termed a widening gap [29, 34, 42].

The presence of seizures in children with CP predicts lower intellectual functioning relative to children without seizures. Most studies, except for data published by Bottcher et al. [43] and van Buuren et al. [29], report a strong association between seizures and lower intellectual functioning [25, 34, 35, 39, 41, 44–46]. A review of the effects of seizure and epilepsy variables on intelligence is beyond the scope of this paper, yet is likely to include seizure frequency, recurrent seizures, age at seizure onset, duration of illness, antiepileptic drugs, type of epilepsy, and EEG findings [47].

In children with CP, FSIQ is likely impacted by underlying motor limitations or secondary impairments in speech and language [25, 30, 48]. Increasing gross motor impairment has been found to be associated with greater cognitive impairment [22, 49, 50]. Exploring the relationship between fine motor and intellectual function, Sherwell et al. [48] found that estimates of intellectual abilities using modified subtests specifically excluding tasks with a substantial fine motor component raised the estimated IQ by approximately 5 points. Using subtests of the Wechsler Intelligence Scale for Children (WISC-III) [51] that requires verbal answers only, Bottcher et al. [43] reported 33 children with spastic CP obtained a mean FSIQ within the average range, of 92.2 (SD 22.8). Stadskleiv et al. [22] attempted to modify tasks to maximise child performances regardless of secondary impairment. The authors assessed 70 children with CP at all GMFCS levels and calculated a composite measure of cognition, using modified response modalities (finger point or eye gaze), depending on child abilities. Using a summary cognitive quotient measure (CQ), significant variability was observed - nearly half had a CQ above 85, 33% obtained a CQ below 70, and 24% were described as having severe adaptive impairment consistent with a diagnosis of ID.

Although general measures of intelligence offer a broad-based assessment of intellectual ability, they are not sensitive to the specific cognitive impairments seen in children with CP [40, 52, 53]. In children with unilateral CP, intelligence can fall within the average range whilst children demonstrate specific cognitive deficits [22, 54]. Therefore, a more detailed examination of cognitive abilities is essential to accurately characterise cognitive development in these children and to allow for exploration of associations between cognition and bimanual performance.

### Specific cognitive deficits in children with cerebral palsy

This study will extend beyond measurement of general intellectual functioning in children with unilateral CP and focus on the impact of attention and higher-level cognitive abilities due to the specific vulnerability of these cognitive areas following early brain injury.

### Executive function

Executive functions (EFs), often referred to as higher-level cognitive functions [55], are defined as those “metacognitive capacities that allow an individual to perceive stimuli from his/her environment, respond adaptively, flexibly change direction, anticipate future goals, consider consequences, and respond in an integrated or common-sense way, utilising all these capacities to serve a common purposive goal” [56]. In line with Alexander and Stuss [57] and Anderson [58], EF has been conceptualised with four distinct domains: (i) attentional control, (ii) information processing, (iii) cognitive flexibility, and (iv) goal setting. EFs are dependent on numerous and complex neuronal systems within the prefrontal cortex and with virtually all other brain regions including the brain stem, occipital, temporal, and parietal lobes, as well as the limbic and subcortical regions [59]. It is the last cognitive function to mature [60] and evolves over a prolonged period of time through brain maturation and life experiences in childhood and adolescence. Each of the four domains of EF has a separate developmental trajectory, yet EF processes operate in an integrative manner.

There are a growing number of studies [22, 33, 43, 53, 61–65] and reviews [38, 66, 67] that have investigated EF in children with CP with findings suggesting difficulties across multiple EF domains. Bottcher et al. [43] report difficulties in all domains of the Behavior Rating Inventory of Executive Function (BRIEF) [68] in a group of 33 children with spastic unilateral and bilateral CP. Bodimeade et al. [53] found children with unilateral CP performed significantly more poorly than typically developing peers on most EF measures, irrespective of side of hemiplegia. Contrary to these findings, Stadskleiv et al. [64] investigated EF in in a population of children with spastic CP and speech/motor impairment. They used a novel test battery comprised of alternative assessment measures and some experimental questionnaires to measure EF and found performances were in the normal range. Further, they investigated the factor structure of the four-domain model of EF described by Anderson et al. [58] and these findings did not support the model of EF proposed by Anderson et al. [58]. However, the validity of these novel measures is unclear.

Bottcher et al. [43] highlights attentional control as a particularly vulnerable area following early brain lesion. The importance of attention as a basic cognitive process has been highlighted by Anderson et al. [69] who propose that attentional processes develop first and influence development of information processing, cognitive flexibility and goal setting. Studies have demonstrated children with CP perform significantly lower than age expectations across a range of attention domains – including focused, sustained, selected and divided attention [43, 53, 70, 71]. Impaired attention may manifest in behaviour such as distractibility and inattention and lead to learning difficulties. While not previously investigated in children with CP, there is a clear rationale that attention difficulties, coupled with an impulsive responding style [33], may impair learning of motor skills that require two hands. While infants and young children achieve innate actions successfully i.e. rolling or walking, without necessarily intending to learn or being aware of what is learned, more complex bimanual tasks require attention and other cognitive processes to guide performance [72]. Evidence from preterm children and children with developmental disorders highlight the influence of attention skills on motor performance. Foulder-Hughes et al. [73] found that movement outcomes were significantly and independently associated with inattentive behaviours in preterm children [73]. Further, attention difficulties are present in up to 50% of children with Developmental Coordination Disorder (DCD) [74], suggesting an association between attention and motor performance.

### Executive function and brain structure

In children with unilateral CP, periventricular white matter damage (PVL) is the most common brain injury, occurring in 36% of children [75]. The integrity of white matter is considered to be important for attention and EF due to the reliance on interconnectivity with other parts of the brain [43]. In PVL, white matter is vulnerable to damage, with the anterior and parietal regions particularly vulnerable. This might act as a common mechanism for motor and cognitive impairment. In addition, periventricular lesions are thought to compromise development of motor pathways [76]. Further, PVL affects the basal ganglia and thalamus [77], which are likely to affect attention and EF [29, 43]. Injury to the basal ganglia and thalami has been found to be associated with lower cognitive [29] and bimanual abilities in children with unilateral CP [9]. This study will provide new insight into the concurrent impact of underlying brain pathology on EF and bimanual performance.

### Executive function and motor planning

Successful motor task performance depends on high-level problem-solving skills such as task initiation, problem solving and sequential ordering [78, 79]. This understanding is central to cognitive-based approaches used to improve task performance in a range of paediatric conditions [79–81]. These approaches promote a verbal-mediated learning experience, where planning and organisational strategies are used to guide successful task performance. Cognitive Orientation to daily Occupational Performance (CO-OP) [82], is the most widely known and uses a verbally-based, problem-solving approach*.* CO-OP has demonstrated excellent efficacy in children with DCD [83, 84]. Emerging evidence for the effectiveness of CO-OP in children with CP [85, 86] suggests the role of higher level cognitive planning and organisation skills in motor performance and a potential avenue for improvement. Surprisingly, the role of planning and organisation deficits (EF) in task performance is yet to be empirically established in children with CP.

Anticipatory action planning or motor planning is essential for skilled task performance and involves the EF domain of goal setting. Specifically, it requires the ability to predict the future state of the motor system, or the consequences of its action [87]. Put simply, it is *knowing what to do, before we do it* [79]. Previous research suggests children with right unilateral CP, secondary to left hemisphere damage, have deficits in anticipatory planning abilities [78]. These children adopt a step-by-step response as the movement progresses rather adopting a planned approach before commencing the task [88]. Deficits in goal setting may, for example, result in a child failing to anticipate the orientation of an object for efficient task performance, or the sequence of movements or direction of force required to complete a task using two hands [81]. Motor planning deficits in children with unilateral CP are observed when a child uses either their more affected or less affected upper limb - suggesting higher order goal setting may be central to motor planning difficulties in children with unilateral CP [78, 88].

Evidence for a relationship between EF difficulties and motor performance is also derived from recent work which demonstrates that complex cognitive and motor skill development continues into early adulthood [89]. This evidence raises important questions about the development of motor functioning in the presence of disrupted acquisition of higher-level cognitive abilities. Preliminary empirical data in typically developing children suggests an important role for aspects of EF in motor performance [90]. The relationship between EF and bimanual performance is yet to be explored in children with CP.

## Methods/Design

### Aim

The broad aim of this study is to examine relationships between EF and bimanual performance, and common neuropathological mechanisms for impaired bimanual performance, to inform existing, and develop new, more effective treatment approaches for children with unilateral CP. Specific aims are:

### Primary aim

1. To examine the association between EF and bimanual performance in children with unilateral CP, whilst accounting for general intellect and unimanual capacity.

### Secondary aims

- To explore the specific associations between sub-domains of EF (attention, impulse control, planning and organising, self-regulation) and bimanual performance.
- To explore relationships between EF, bimanual performance and the type and severity of brain injury (using MRI classification) in children with unilateral CP, and to identify risk factors for concurrent cognitive and motor impairment.

### Hypothesis

#### Primary Hypothesis

In children with unilateral CP, greater EF impairment will be associated with concurrent impairments in bimanual performance, when general intellectual function and unilateral function are taken into account.

#### Secondary Hypotheses

In children with unilateral CP, difficulties in specific executive abilities (attention control, cognitive flexibility, speed of processing and goal setting) will be associated with poorer bimanual performance. Specific EF deficits are expected to underlie the broad EF impairment and explain the mechanism for non-motor difficulties experienced by children with unilateral CP in completing bimanual tasks.

In children with unilateral CP, EF and bimanual performance are influenced by common underlying brain pathology (e.g. pathophysiological process/site and severity of brain lesion). Although the relationship between imaging findings and cognitive and motor functioning is yet to be established [38], in this study we expect to gain evidence that the relationship between EF and bimanual performance outcomes are influenced by the integrity of underlying neuroanatomical structures. Precise relationships may vary depending on the type and timing of brain lesion.

### Design

This study will use a prospective, cross-sectional observational design to examine the association between and among EF, type and severity of brain injury and bimanual performance in a sample of children, aged 6 to 12 years, with unilateral CP.

### Participants

#### Inclusion Criteria

Children will be eligible to participate if they have a confirmed diagnosis of unilateral CP as reported in the medical history by a medical specialist (i.e. neurologist, paediatrician), are aged 6 to 12 years at the time of assessment, present to one of five participating sites (between July 2012 and August 2015), and have sufficient cooperation and language skills to complete the assessments.

#### Exclusion Criteria

Children will be excluded from the study if they have had upper limb surgery within 12 months of assessment or upper limb injection of Botulinum toxin-A within 3 months of assessment. There will be no criteria relating to exclusion of children/parents due to language spoken other than English, presence of co-morbidity or socio-economic status.

### Ethics and dissemination

Prior to study commencement, a multi-institute research agreement was signed between each participating study site and the lead site, Monash Children’s Hospital. Ethical approval has been received from Monash Children’s Hospital, Victoria (HREC: 12167B); The Royal Children’s Hospital, Victoria (HREC: 32232A); Cerebral Palsy Alliance, NSW (HREC: 2012–12-03); Lady Cilento Children’s Hospital, Queensland (HREC/12/QRCH/218) and Princess Margaret Hospital, Western Australia (HREC: 2013062). Parents or guardians of all participants will provide informed written consent for their child to take part in the study. Following assessments, parents will receive a brief neuropsychological report detailing the results of child cognitive assessment and general strategies to assist any identified cognitive weaknesses. If required, referral to appropriate clinical services will be provided, including clinical neuropsychology or mental health clinicians. Modifications to the protocol will be reported to each Human Research Ethics Committee by Site Investigators and noted on the trial registry by the Chief Investigator.

Upon entry to the study, each child will be assigned a unique study code by the site investigator. All paper and videotaped assessments will be labelled using the unique study code. No identifying information will be attached. Data will only be re-identifiable to the local site. The Chief Investigators (BH, CI) and other investigating sites will not be able to identify data collected from other participating sites.

The results of this study are scheduled to be published in peer reviewed publications and will be presented at national and international conferences. The minimum requirement for authorship will be in accordance with the Vancouver Protocol [91].

### Sample size

A power analysis was conducted and it revealed a sample size of 150 children is sufficient to detect a minimum effect size of 0.12 (Cohen’s ƒ2) with 10 predictors at 80% power and a probability level of 0.05.

### Study setting and recruitment

Children will be identified from five tertiary paediatric treatment centres across Australia. Children will be recruited from: Monash Children’s Hospital, Victoria; Royal Children’s Hospital, Victoria; Cerebral Palsy Alliance, New South Wales; Lady Cilento Children’s Hospital, Queensland and; Princess Margaret Hospital, Western Australia. The lead investigator, or HREC approved delegate at each trial site will identify potential participants and provide written and verbal information about the study to potential families. Eligibility will be determined through discussion with parents and/or review of medical files. The first child was enrolled in this study on 5th December 2012. Data collection was completed for 131 children on 7th October, 2015.

### Data collection

#### Demographic Data

Basic demographic and clinical information will be collected from families and health records and documented using a using a standardised clinical record form. Information includes: age, diagnosis, gender, gestational age, birth weight, co-morbidities, current schooling and integration assistance, family makeup, medication, seizure history and previous MRI.

#### Clinical Assessment

Children will be assessed within six weeks of recruitment across one or two appointments. A detailed study assessment manual developed by the Chief Investigators (BH, CI) will provided to each site. Clinical assessment will be undertaken by occupational therapists, neuropsychologists or supervised psychologists, all educated in study aims and assessment protocols. Experienced paediatric radiologists will code for lesion characteristics. The radiologist will be blinded to the results of the clinical assessments. The clinical assessors will be blinded to the type of brain lesion.

#### Classification measures

For descriptive purposes, children’s functional level will be classified using the Manual Ability Classification System (MACS) [92], Gross Motor Function Classification System (GMFCS) [93] and Communication Function Classification System (CFCS**)** [94].

#### Upper Limb Motor Performance

##### Primary

**Assisting hand assessment**

The Assisting Hand Assessment (School Kids Version 5.0) will be used to measure bimanual performance (AHA) [95, 96]. The AHA is a standardized, criterion-referenced test for children aged 18 months to 12 years, who have unilateral upper limb impairment. It aims to measure how effectively a child uses their affected hand in bimanual play activities. The AHA will be conducted by video observation of the child using specific toys of the AHA test kit to play one of two board games. The 20 items define different actions and will be scored by a certified AHA rater (occupational therapist) on a 4-point rating scale. The sum of raw scores (sum score) varies between 20 (low ability) to 80 (high ability). Raw scores are converted to interval level data using Rasch analysis. Rescaled logit-based AHA units ranging from 0–100 will be used for data analysis [95]. A higher number indicates higher ability. The psychometric properties of the AHA have been described in several studies [95, 97–99].

##### *Secondary*

**Box and blocks test**

The Blocks and Box Test aims to measure manual dexterity by having a child move as many 2.5 cm blocks as they can from one side of a box, over a low partition to the other side of the box, one-by-one, in one minute [100]. The Box and Blocks Test has age norms for children aged 3 to 10 years [101] and 6 to 19 years [102]. Test-re-test reliability coefficients for adults is reported as r = 0.976 for the right hand, and r = 0.937 for the left hand [100].

ABILHAND-Kids

The ABILHAND-Kids is a questionnaire about manual ability in self-care activities in children with upper limb impairment [103]. The scale consists of 21 mostly bimanual items rated by parents as impossible, difficult, easy to complete or unknown. Scores on the ABILHAND-Kids are significantly related to school education, type of CP, and gross motor function but not age, gender or handedness [104]. Its range and measurement precision are appropriate for clinical practice (reliability: r = 0.94; reproducibility over time: r = 0.91) [103].

Anticipatory action planning

The Sword Test will be used to measure anticipatory action planning [105]. Children will be asked to use their dominant hand to grasp the handle of a plastic sword that has been placed randomly in one of six positions on a template board. Children are required to place the sword into a hole in a wooden block. Some of the sword positions on the template are control positions and others are “critical” positions and require children to plan and make an adjustment to the way they approach and grasp the sword so it is inserted in the hole in a position of end comfort. The dependent variable is whether the posture of the hand at the end of the action is comfortable. For data analyses the proportion of comfortable end postures in the critical conditions and the control conditions will be used. Thus, for every child there are two scores, an average for the critical conditions and an average for the control conditions. Test–retest and inter-rater reliability for the Sword Test is excellent, with an intraclass correlation coefficient (ICC) score of .90 and .95 respectively [106].

#### Cognitive outcomes

##### *Primary*

**Executive function**

**Fig. 1:**
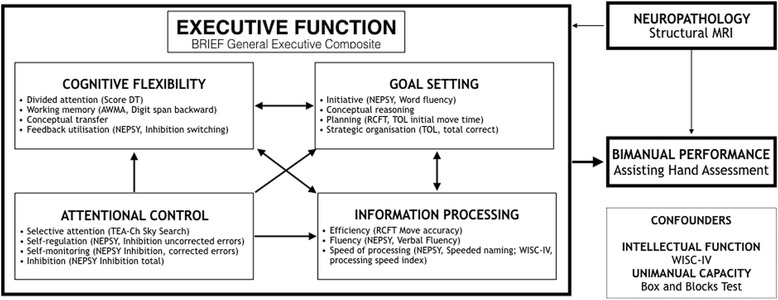
Proposed model of executive function, bimanual performance and neuropathology with corresponding assessments

We have schematically mapped each of the assessment tasks to Anderson’s [58] four-domain model of child EF, demonstrated in Fig. 1. In this model, the four domains are attention control (which includes selective attention, self-regulation, self-monitoring, and inhibition), information processing (efficiency, fluency, and speed of producing) cognitive flexibility (the ability to divide attention and shift between response sets, complex span/working memory and feedback utilisation), as well as a goal setting component (including efficient idea generation, as well as the ability to initiate and plan goals). In this study, we will also include a broad measure of overall EF that subsumes all of the four subdomains.

As an overall composite measure of EF, the Behaviour Rating Inventory of Executive Function (BRIEF) – Parent Version [68] will be used to obtain parent ratings of current functional, emotional and behavioural manifestations of executive dysfunction. This is an 86-item questionnaire, valid for use in children 5–18 years. T-scores provide information on functioning relative to the standardised sample (mean = 50, standard deviation = 10). Two indexes will be used for analysis. The overall General Executive Composite Score will be the primary measure. A higher score indicates a higher level of executive dysfunction, with a score above 65 indicating abnormal EF. The Metacognition Index and Behavioural Regulation Index will be the secondary outcome. Higher scores indicate greater difficulties with EF.

*(a) Attention contro*

Attention control abilities will be assessed using the Test of Everyday Attention for Children (TEA-Ch) [107]. The TEA-Ch comprises nine subtests, and is valid for children 6–16 years. The scaled scores have a mean = 10 and standard deviation = 3. Attention control will be assessed using the Sky Search task. Children will be given an A3 sheet with rows of space-craft and asked to find all targets (an identical pair of space craft). Children mark a box when they are finished. Total number of correct targets and time taken will be recorded.

*Self-monitoring, self-regulation and inhibition* will be measured using the Inhibition task of the NEPSY-II, designed for children 5–16 years. Children are screened for basic naming of included shapes, and then are asked to look at a series of black and white shapes or arrows and name either the shape, direction, or an alternative response, depending on the colour of the shape or arrow. Inhibition of automatic responses to stimuli is required for successful task completion. Scaled scores are calculated (mean = 10, standard score = 3) using age-based normative data for each of the described areas.

*(b) Cognitive flexibility*

*Divided attention* will be measured using the Sky Search DT task from the TEA-Ch. This requires a test score from the SCORE task also. In the Score! Task, children are first asked to count to 10 as a screen. Then, children continuously count tones for 10 items (in which tones are separated by silent inter-stimulus intervals of variable duration between 500 and 5000 msec). Counting to 15 will be tested as a pre-requisite for this task. In Sky Search DT, children are required to complete the Score! and the Sky Search tasks simultaneously. Decrements in task performance relative to performances on isolated tasks will be computed as a measure of attention decrement under dual-task conditions.

*Working memory* will be assessed using tasks from the Automated Working Memory Assessment Visuospatial (AWMA) [66]. The AWMA is a computer administered test valid for use in children and adults (4–22 years). Children will complete two tasks tapping visuospatial short term (Dot Matrix, Block Recall) and two visuospatial working memory tasks (Mr X, Backwards Dot Matrix).

*Feedback utilisation* will be measured using the Inhibition Switching Task and scores from the NEPSY II. This task builds on the Inhibition naming task, and includes an attentional switching component, providing naming accuracy and speed scores.

*(c) Speed of information processing*

*Efficiency* and *fluency* will be calculated using specific test scores from the Word generation/fluency subtest from the NEPSY-II [108] as well as the Tower of London (TOL) task [109]. The NEPSY-II word generation/fluency task is designed for use in children 3–16 years, and provides age based scaled scores (mean = 10, SD = 3). Children will be required to rapidly name words based on semantic and phonemic (initial letter) cues.

The TOL task involves children completing 12 ‘problems’ in which they are required to rearrange three coloured balls on posts so that a new configuration corresponds to the pattern presented on a stimulus card. Normative data from Anderson et al. [110], will be used to transform raw scores into age based standard scores (mean = 100, SD = 15). Efficiency will be calculated using the TOL move accuracy ratio.

Fluency will be calculated using the total number of words generated in the NEPSY-II word generation task (including errors).

Basic *speed of processing* will be measured using the processing speed index, calculated from the WISC-IV. In order to provide a measure of processing speed independent of fine motor function, the Speeded Naming task from the Neuropsychology Assessment for Children-II (NEPSY-II) [108] will also be used.

*(d) Goal setting*

Goal setting includes a number of sub components. Initiative is the ability to develop new concepts, measured by the total number of correct words in the word generation/fluency subtest from the NEPSY-II [108]. The total time required to plan the first move on the TOL provides a measure of planning, using additional process scores calculated according to guidelines provided by Anderson et al. [110]. The TOL total correct provides a measure of strategic organisation. The Rey Complex Figure Test (RCFT) assesses a child’s ability to strategically organise their response to a complex problem. Strategic organisation scores will be calculated as per Anderson et al. [111].

##### *Secondary*

**General intellectual ability**

General intelligence will be assessed using the Wechsler Intelligence Scale for Children (4th Ed) (WISC-IV) [112]. The test comprises of ten core (and five supplemental) subtests used to calculate the FSIQ. It is valid for children aged 6–16 years. Verbal comprehension (VC), perceptual reasoning index (PRI), working memory and processing speed will be calculated as per the WISC-IV manual. Index scores have a mean = 100 and standard deviation = 15. If children are unable to complete subtests due to fine motor impairment, language impairment or reduced general ability, a composite score will be calculated [113]. For children with insufficient skills to complete subtests comprising the VC or PRI index, the alternative index will be used, so as not to disadvantage children with varying motor/language profiles. Where insufficient subtests are available, the index-based seven subtest short form score will be calculated [114]. For children unable to complete sufficient subtests to calculate a VC or PRI, this will be interpreted as profound intellectual disability, an IQ standard score of −4 standard deviation (SD) will be assigned.

*Functional attention* in daily tasks will be measured using the Conners 3rd Edition [115], a 99-item rating scale of attention related behaviours, valid for children aged 6–18 years. Age-referenced T-scores are provided (mean = 50, SD = 10). It has a large representative normative sample and is strongly connected to the Diagnostic and Statistical Manual of Mental Disorders-IV diagnostic criteria for attention disorders.

#### Neuropathology

We will obtain consent from participants’ parents/guardians to access existing brain magnetic resonance imaging (MRI) scans if they are available. Scans taken before the age of 18 months will only be included if the type of pathology was obvious and more recent imaging was not expected to provide further information. Two highly experienced paediatric radiologists (MD, JB) will assess the images to document the location, type and severity of brain injury according to the method developed by Leonard and colleagues [116, 117].

### Statistical considerations

Data entry will be undertaken by the Chief Investigators (BH, CI) and research assistants. All statistical analyses will be led by a biostatistician and the Chief Investigators in consultation with the research team. Stata Statistical Software version 15 will be used for analysis. Statistics will be reported with 95% confidence interval, where appropriate, and 5% level of significance will be used.

Data will be analysed using structural equation modelling (SEM). Given the complexity of the model, confirmatory factor analysis (CFA) will be initially conducted on the four elements of EF (cognitive flexibility, goal setting, attention control and information processing) to ensure that three or four chosen measures of each element correlate well and measure the selected element. Regression based-factor scores from the CFA will then be used in the subsequent SEM that incorporates the variables in the model, presented in Fig. 1. The SEM will be used to examine the direct and indirect relationships among between EF (estimated using the BRIEF questionnaire and also the latent variable estimated using the four elements of EF). The model will be adjusted for child characteristics, unimanual capacity and intellectual functioning. For both CFA and SEM, goodness of fit of the final models will be assessed using the chi-square test statistic and goodness of fit indices, particularly the Root Mean Square Error of Approximation (RMSEA), goodness-of-fit index (GFI), Tacker-Lewis index (TLI) and the comparative fit index (CFI). Low levels of missing data are anticipated therefore no missing data imputation is planned and the analyses will explore the appropriateness of using the either maximum likelihood (ml) or maximum likelihood with missing values (mlmv) estimators.

## Discussion

This study protocol reports a prospective, multi-centre, cross-sectional study that will provide new knowledge of the multiple and concurrent processes that contribute to task performance in children with unilateral CP. Cognition and bimanual performance are both factors known to affect functional outcomes but have not yet been studied concurrently. Knowledge from this study has the capacity to significantly influence, inform and adapt clinical practice. It is anticipated that distinct profiles of motor, cognitive and neuropathology will be identified. This understanding will improve the specificity and targeting of existing upper limb interventions. It may also lead to the development of novel methods to improve cognitive and motor outcomes in children with unilateral CP. Dissemination of results and translation of knowledge into clinical practice will begin immediately via professional development opportunities provided by site investigators. The large number of university affiliations across the study team also provides opportunities to integrate learning into undergraduate education. Knowledge translation strategies will also target parents and teachers of children with CP.

## Abbreviations

AHA: Assisting Hand Assessment
AWMA: Automated Working Memory Assessment
BRIEF: The Behaviour Rating Inventory of Executive Function
CFCS: Communication Function Classification System
CO-OP: Cognitive Orientation to daily Occupational Performance
CP: Cerebral Palsy
CQ: Cognitive Quotient
DCD: Developmental Coordination Disorder
EF: Executive Functioning
FSIQ: Full-Scale Intellectual Quotient
GMFCS: Gross Motor Function Classification System
ICC: Intraclass correlation coefficient
ID: Intellectual Disability
IQ: Intellectual Quotient
MACS: Manual Ability Classification System
MRI: Magnetic Resonance Imaging
NEPSY-II: Neuropsychology Assessment for Children-II
PAIS: Perinatal Arterial Ischaemic Stroke
PRI: Perceptual Reasoning Index
PVHI: Periventricular Haemorrhagic Infarction
PVL: Periventricular White Matter damage
SD: Standard Deviation
TEA-Ch: Test of Everyday Attention for Children
TOL: Tower of London
VC: Verbal Comprehension
WISC-IV: Wechsler Intelligence Scale for Children 4th Edition
